# Normative values for the physical activity scale for the elderly in community-dwelling men and women 45 to 85 years old: an analysis from the CLSA

**DOI:** 10.1186/s12966-025-01820-w

**Published:** 2025-10-14

**Authors:** Cassandra D’Amore, Alexandra Mayhew, Lauren E. Griffith, Parminder Raina, Julie Richardson, Marla K. Beauchamp

**Affiliations:** 1 School of Rehabilitation Science, McMaster University, 1400 Main St. W., IAHS - Room 403, Hamilton, ON L8S 1C7 Canada; 2 Department of Health Research Methods, Evidence, and Impact, McMaster University, 1280 Main St W. MIP- Room 309, Hamilton, ON L8S 4K1 Canada

**Keywords:** Aging, Canadian Longitudinal Study on Aging, Physical activity (PA), Reference values, Questionnaire

## Abstract

**Background:**

Monitoring and improving physical activity levels is essential for promoting healthy aging. The objective of this study was to create age-specific normative values for the Physical Activity Scale for the Elderly (PASE) among community-dwelling women and men aged 45–85 years old.

**Methods:**

36,701 participants (47% female) aged 45–85 years old, free of any mobility limitation or activities of daily living disability from the Canadian Longitudinal Study on Aging (CLSA) were included. Best fitting models were identified using Generalized Akaike Information Criteria values and cross-validation. Seasonal differences for males and females were also explored.

**Results:**

Separate models for males and females are presented, providing a range of percentile values (5–95%) in charts and tables. Total PASE scores were highest in 45-year-olds and decreased with age. Seasonal differences were not substantial or consistent at the population level.

**Conclusions:**

The age- and sex- specific normative values provided can improve the interpretability of PASE scores among middle-aged and older adults. In addition to PA guideline cut-offs, normative values provide further information for monitoring physical activity by allowing for more personalized observations that account for healthy variation.

**Supplementary Information:**

The online version contains supplementary material available at 10.1186/s12966-025-01820-w.

## Introduction

Physical activity has been highlighted by the World Health Organization (WHO) for its potential to play a pivotal role in achieving healthy aging for many older adults [1]. Physical activity (PA) is commonly defined as any bodily movement produced by skeletal muscle that requires energy expenditure [2]. Higher levels of PA are associated with improved trajectories of aging [3]. Despite what we know, there are persistently high levels of inactivity worldwide, costing billions of dollars annually in preventable healthcare costs [4]. Improved methods of measuring and monitoring PA behaviour are essential for curbing the tide of low PA and promoting better health in older ages.

A popular indicator of insufficient PA is the cut-off value provided by international PA guidelines for people over the age of 18 (i.e., 150 min of moderate to vigorous PA a week) [5]. While activity levels in keeping with this guideline are associated with numerous positive health outcomes, use of a single threshold for PA does not allow for variation among individuals that may reflect preferences and values. Additionally, for monitoring PA levels and changes over time, use of the guideline cut-off does not capture smaller increases in PA, which have shown positive health benefits; [6] a concept that is currently being promoted in many countries (i.e., “some is better than none”) [5]. Normative values may help improve monitoring of PA behaviour by allowing for variation across ages but still signalling when intervention is needed. Normative values provide a reference based on a ‘healthy’ population that allows for comparison to a standard and can be used to indicate that something is amiss or in need of intervention. For example, child and adolescent height and weight growth curves are commonly used for this purpose [7]. Among older adults, normative values for PA could provide more detailed information on PA behaviour that can be used to promote healthy aging and assist clinicians and researchers to interpret scores on commonly used PA outcome measures.

The Physical Activity Scale for the Elderly (PASE) was originally developed to capture a representative picture of PA in older ages. The PASE has been recommended for use in community-dwelling older adults as well as those with multiple chronic conditions compared to other self-report PA questionnaires (e.g., International Physical Activity Questionnaire (IPAQ), Community Healthy Activities Model Program for Seniors (CHAMPS)) due to its reliability and its ability to capture lighter intensity activities [8, 9]. Light intensity activity is associated with positive health outcomes on its own and in addition to achieving 150 min of moderate to vigorous PA [10]. The PASE has been widely used in research with over 2,000 citations for the seminal 1993 Washburn paper (reverse search from Web of Science). Researchers from 35 countries have used the PASE, and at least eight validated translated versions have been developed (e.g., Arabic [11], Chinese [12], Italian [13], Malay [14], Norwegian [15], Persian [16], Polish [17], and Turkish [18]) [19]. The popularity and psychometric evidence for the PASE provide a strong rationale for creating normative values that would increase the interpretability of PASE scores for clinicians and researchers.

Therefore, the objective of this study was to create age- and sex-specific normative values for the PASE total score among community-dwelling adults aged 45–85 years using baseline data from the large population-based Canadian Longitudinal Study on Aging (CLSA). Seasonal changes have shown to impact PA activity but it is unclear if there are other factors influencing this relationship (e.g., health conditions, age) [20, 21]. Therefore, we also stratified for season to explore its impact on PA levels for different ages of men and women [20]. 

## Methods

### Study population description

The CLSA is a longitudinal population-based cohort study. Baseline data collection was completed in 2015 and included 51,388 Canadians between the ages of 45 and 85 years from across all 10 provinces [22]. The CLSA consists of two samples, the tracking cohort (*n* = 21,241), provided data via telephone interviews and the comprehensive cohort (*n* = 30,097) that is completed through a combination of telephone, in-home, and site visit assessments. Persons living in the Canadian territories, on Federal First Nations reserves and other provincial First Nations settlements, or long-term care or institutions, as well as full-time members of the Canadian Armed Forces and persons presenting with cognitive impairment at the time of recruitment were not included. Additionally, participants from the comprehensive cohort were recruited from a restricted area of 25–50 km around one of the 11 data collection sites, located in seven provinces. Detailed sampling methods are described elsewhere [23]. 

Our analysis used data collected from CLSA baseline. The aim of this study is to create normative values; therefore, we applied exclusion criteria to create a sample that would represent the ‘healthy’ population in the context of PA [7]. To capture a sample free of PA restrictions we only included individuals free of any mobility limitations. We did this by excluding individuals who reported use of a gait aid (i.e., cane/walking stick, walker, wheelchair, or motorized scooter). We also excluded anyone who indicated that they needed assistance with any of the 14 activities of daily living (ADL) or instrumental activities of daily living (IADL) from the Older Americans Resources and Services Multidimensional Assessment Questionnaire as part of baseline questionnaires in the CLSA [24]. No other exclusion criteria were applied to the original CLSA sample.

### The physical activity scale for the elderly (PASE)

In the CLSA the PASE was completed during the maintaining contact questionnaire via telephone interviews. The PASE was created by Washburn et al. in 1993 to more accurately reflect older adults PA compared to age neutral questionnaires [25]. The PASE uses 10 questions to ask participants about activities they have completed over the last 7 days [25, 26]. Unlike age-neutral (i.e., 18 + years old) PA questionnaires like the IPAQ or Global Physical Activity Questionnaire, which focus on moderate to vigorous PA, the PASE captures all intensity levels (i.e., light to vigorous) allowing us to examine total PA. Types of activities include walking outside the home, light, moderate, and strenuous sport and recreation, exercise, housework (indoor and outdoor), caring for another person, and work/volunteer (made up of standing or walking). The amount of time spent in activities is collected (e.g., duration and frequency) for all except household activities which are dichotomous yes/no. These responses along with the predetermined weights for each activity are used to calculate a total PASE score. The activity weights were created by Washburn et al. (1993) using 3-day tracking with a movement counter, activity diary and a global self-reported activity item [25]. Scores on the PASE range from 0 to over 400, with higher scores representing greater activity levels. The PASE also collects information on sedentary activity including a question on sitting activities and one on level of work/volunteering (description: mainly sitting with some standing) that do not contribute to the total score and was not used in this analysis.

#### Psychometric properties of the PASE

The PASE is recommended for use in older adults with good reliability across versions (test-retest intraclass correlations ranging from 0.60 to 0.98) [9, 11]. The PASE has been validated using other PA measures including the self-report International Physical Activity Questionnaire (*r* = 0.69) [16], Japan Arteriosclerosis Longitudinal Study PA questionnaire (*p* = 0.48) [9], and direct measures such as accelerometers (energy expenditure (*p* = 0.53) [14], walking steps (*p* = 0.39) [14], and activity counts (*p* = 0.36–0.43) [9]. Construct validity for the PASE as a measure of PA has been demonstrated by associations with functional physical performance measures including the Timed Up and Go (*r*=−0.45 [11] to *r*=−0.69 [16]), balance (*r* = 0.54 [27] to *p* = 0.55 [12]), Five Time Sit to Stand test (*p*=−0.28 [12] to *r* = 0.56 [27]), and walking (10 m *p*=−0.28, 6 min *r* = 0.68) [9]. As well as questionnaires including the Short Form 36 (physical function *p* = 0.58 [12]), Activities of Daily living (*r* = 0.78 [16]), and Lawton Instrumental Activities of Daily Living Scale (*r* = 0.42 [27, 28]).

### Statistical analyses

Analyses were stratified by self-reported sex at birth. In addition, we explored the effects of seasonal differences on PA through stratifications to account for the effect of temperatures and weather on activity level [20]. Season stratifications were based on the date participants completed their PASE questionnaires as part of the maintaining contact questionnaire and divided into four groups: Winter (January-March), Spring (April-June), Summer (July-September), and Fall (October-December). All analyses were completed using RStudio Team (version 4.2.3, 2020, PBC, Boston, MA). The CLSA provides survey sampling weights, these were applied for the descriptive and modeling analyses (inflation and analytic weights, respectively). The validity of the exclusion criteria was examined by comparing total PASE score for those included versus excluded with independent t-tests in each 10-year age band (45–54, 55–64, 65–74, 75+).

Normative values were created using the methods developed by Mayhew et al. (2023), which are described in detail elsewhere [29]. Briefly, two approaches were used to identify the best fitting model: General Additive Models for Location Scale and Shape (GAMLSS) and quantile regression-based models. To identify the best fit GAMLSS model different combinations of distributions, smoothing techniques and penalty for degrees of freedom were tested and ranked using Generalized Akaike Information Criteria values (GAIC) (‘*gamlss*’ package in R). For quantile regression the following models were fitted to the data (‘*quantreg*’ package): (i) age as a linear predictor, (ii) polynomial functions (to the order of 2–4), (iii) smoothed quantile regression (‘*arqss*’ package), and (iv) fractional polynomial.

To compare the best fitting GAMLSS and the quantile regression models sex-stratified cross-validation was performed. 70% of our sample was randomly selected from each year of age for males and females to create training datasets to develop models. The models developed were then applied to the remaining 30% of participants to estimate the percentage of participants that were below designated percentiles. This process was repeated 100 times for each model and the average estimates below the 5th, 50th and above the 95th percentiles were used to assess how well models fit the data. The same steps were completed with stratification for season. Research ethics approval was received from the Hamilton Integrated Research Ethics Board (application # 7342).

## Results

### Participant characteristics

Of the 51,338 participants in the CLSA, 11,601 (22.60%) reported using a gait aid or needing assistance with at least one activity of daily living or instrumental activity of daily living and were excluded from this analysis. Additionally, 3,036 (5.91%) participants were removed due to missing details on the inclusion criteria or missing/incomplete PASE data. The remaining 36,701 participants were included in this analysis, with a mean age of 61.63 (SD 9.94); 47% were female (Supplemental Table A1). Mean PASE scores were found to be significantly lower for those excluded from our sample compared to included participants for males and females (*P* < 0.001, Supplemental Table B1).

**Fig. 1:**
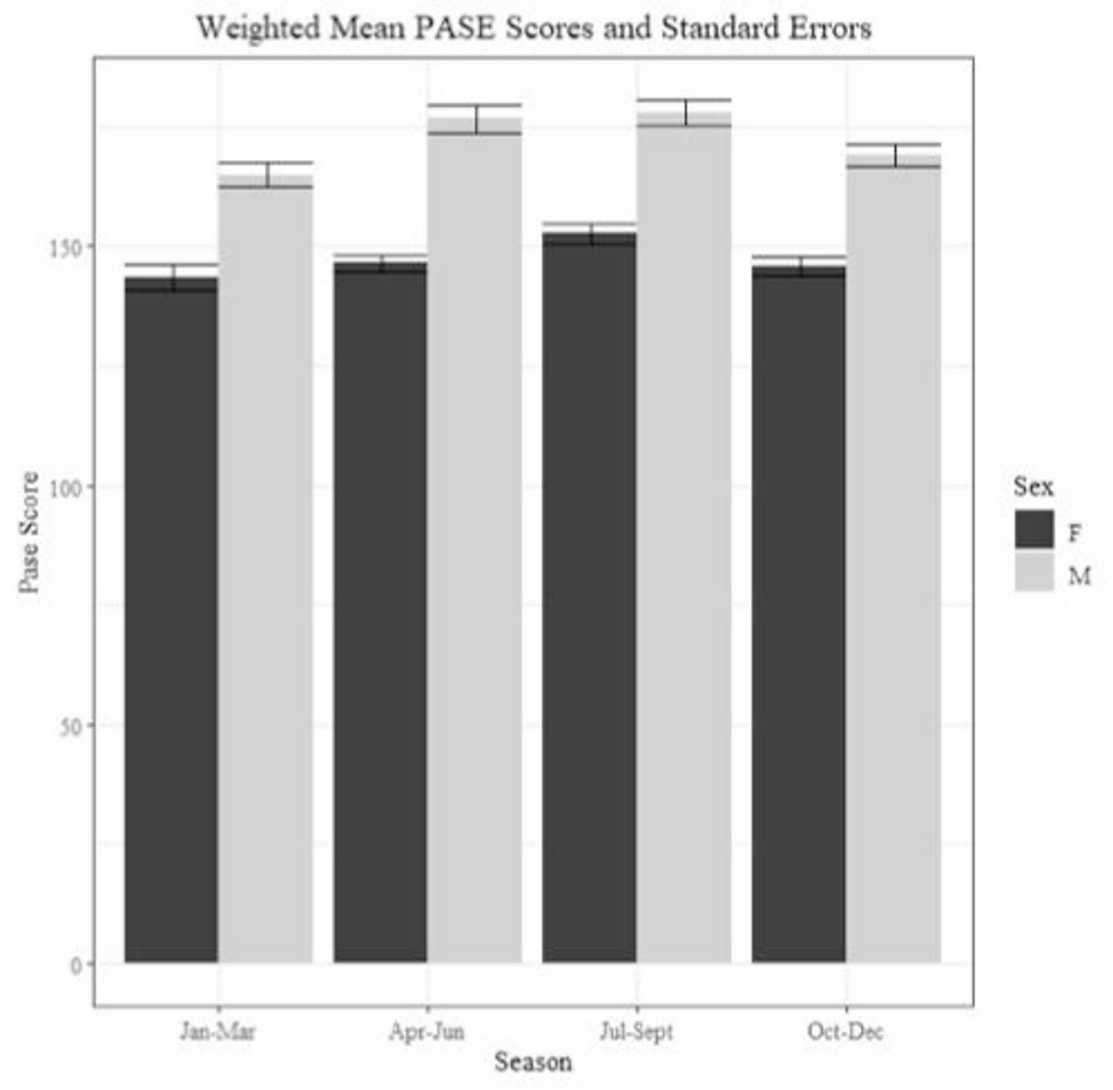
Total PASE scores for females and males across seasons

The included sample of 36,701 participants represented a target population of over 9 million Canadians, we included a descriptive analysis of the weighted sample stratified by sex in Table 1. Overall, close to 50% of the target sample was female, 73% used at least one medication, greater than 50% had self-reported very good or excellent health, 60% had a post-secondary degree, and 80% resided in an urban area. Physical activity measured by the PASE was captured equally across all four seasons, with 70% of participants agreeing their PA levels at the time of assessment were representative of the last 12 months (Fig. 1). Mean PASE scores were 172 (SD 82.47) and 147 (SD 71.89) for males and females respectively.

**Table 1:** Weighted demographic characteristics

	Weighted		
	Total *n* = 9,623,206	Male *n* = 4,975,812 (51.71%)	Female *n* = 4,647,394 (48.29%)
**Age**	58.72 (9.72)	58.78 (9.78)	58.65 (9.66)
**Cultural/racial background** European Non-European Multiple origins	9,107,471 (95%) 403,977.8 (4%) 102,278.5 (1%)	4,703,957 (95%) 216,788 (4%) 46,877 (1%)	4,403,514 (95%) 187,190 (4%) 55,402 (1%)
**Marital status** In a relationship Widowed Divorced/separated Single	7,477,748 (78%) 565,873 (6%) 853,398(9%) 600,452 (6%)	4,116,058 (83%) 134,9(98 (3%) 354,139 (7%) 311,506 (6%)	3,361,691 (72%) 430,875 (9%) 499,259 (11%) 288,946 (6%)
**Had a fall in the previous 12 months** (Yes)	904,903 (9%)	399,648 (8%)	505,255(11%)
**Diagnosed with a chronic medical condition** Cancer Cardiovascular Diabetes Mental health Musculoskeletal Neurological Respiratory Vision	1,084,084 (11%) 3,656,924 (38%) 1,356,175 (14%) 1,619,806 (17%) 4,430,093 (46%) 460,982 (5%) 1,360,350 (14%) 1,864,626 (19%)	503,147 (10%) 203,457 (40%) 768,008 (15%) 672,750 (14%) 2,101,505 (42%) 267,791(5%) 651,852 (13%) 854,158 (17%)	580,937 (13%) 1,622,348 (35%) 588,167 (13%) 947,056 (20%) 2,328,589 (50%) 193,191 (4%) 708,498 (14%) 1,010,468 (22%)
**Number of medications** None One Two 3+	2,600,702 (27%) 1,850,600 (19%) 1,413,448 (15%) 3,708,847 (39%)	1,466,868 (29%) 945,439 (19%) 707,812 (14%) 1,828,983 (37%)	1,133,834 (24%) 905,161 (19%) 705,636 (15%) 1,879,864 (40%)
**Smoking behaviour** Current smoker Former smoker Never smoked	1,114,849 (12%) 5,689,048 (59%) 2,773,922 (29%)	563,284 (11%) 3,147,746 (63%) 1,246,986 (25%)	551,565 (12%) 2,541,302 (55%) 1,526,936 (33%)
**Self-reported general health** Excellent Very good Good Fair Poor	1,996,538 (21%) 3,956,889 (41%) 2,922,902 (30%) 637,339 (7%) 102,945 (1%)	958,400 (19%) 1,990,296 (40%) 1,607,883 (32%) 359,905 (7%) 57,242 (< 1%)	1,038,138 (22%) 1,966,593 (42%) 1,315,020 (28%) 277,434 (6%) 45,702 (< 1%)
**Education** Less than secondary school graduation Secondary school graduation Some post-secondary education Post-secondary degree/diploma	1,649,690 (17%) 1,350,630 (14%) 855,603 (9%) 5,740,628 (60%)	854,543 (17%) 628,374 (13%) 445,586 (9%) 3,031,277 (61%)	795,146 (17%) 722,256 (16%) 410,016 (9%) 2,709,352 (58%)
**Household income** less than $20,000 $20,000 or more, but less than $50,000 50,000 or more, but less than $100,000 $100,000 or more, but less than $150,000 $150,000 or more Missing	395,365 (4%) 2,114,108 (22%) 3,379,029 (35%) 1,805,244 (19%) 1,470,916 (15%) 458,543 (5%)	154,630 (3%) 988,459 (20%) 1,808,910 (36%) 997,360 (20%) 850,865 (17%) 175,589 (4%)	240,735 (5%) 1,125,649 (24%) 1,570,119 (34%) 807,884 (17%) 620,052 (13%) 282,954 (6%)
**Type of dwelling** House (detached, semi-detached, duplex or townhouse) Apartment or condominium Other	8,440,833 (88%) 1,077,120 (12%) 104,966 (1%)	4,393,994 (88%) 529,972 (11%) 50,440 (1%)	4,046,838 (87%) 547,148 (12%) 53,351 (1%)
**Classified as living in rural area**	2,067,563 (21%)	1,026,597 (21%)	1,040,965 (22%)
**Greenspace** (Normalized difference vegetation index) Mean of NDVI within 500 m	0.43 (0.13)	0.43 (0.13)	0.42 (0.13)
**Neighborhood safety** Local area is kept very clean (agreed) People would be **afraid** to walk alone after dark in local area (agreed) Vandalism or graffiti are a big problem in local area (agreed)	9,212,306 (96%) 941,157 (10%) 561,165 (6%)	4,745,590 (95%) 363,056 (7%) 302,910 (6%)	4,466,716 (96%) 578,520 (12%) 258,255 (6%)
**Physical activity (Total PASE score)**	160.41 (78.55)	172.57 (82.47)	147.39 (71.89)
**Physical activity represent last 12 months** Agreed Neither Disagreed	6,723,075 (70%) 203,556 (2%) 2,669,404 (28%)	3,578,708 (72%) 112,096 (2%) 1,270,941 (26%)	3,144,367 (68%) 91,460 (2%) 1,398,463 (30%)
**Season of physical activity measurement** Winter (Jan-Mar) Spring (Apr-Jun) Summer (Jul-Sept) Autumn (Oct-Dec)	1,860,490 (19%) 2,551,054 (27%) 2,503,782 (26%) 2,707,880 (28%)	994,015 (20%) 1,337,623 (27%) 1,287,546 (26%) 1,356,629 (27%)	866,475 (19%) 1,213,431 (26%) 1,216,236 (26%) 1,351,252(29%)

NDVI metrics, indexed to DMTI Spatial Inc. postal codes, were provided by CANUE (Canadian Urban Environmental Health Research Consortium).

*Table References*: **1**.USGS Landsat 5 TM TOA Reflectance (Orthorectified), 1984 to 2011, accessed July 2017, from https://explorer.earthengine.google.com/#detail/LANDSAT%2FL T5_L1T_TOA; **2**.USGS Landsat 8 TOA Reflectance (Orthorectified), 2013 to 2017, accessed July 2017, from https://explorer.earthengine.google.com/#detail/LANDSAT%2FL C8_L1T_TOA; **3**.Landsat 5 TM Annual Greenest-Pixel TOA Reflectance Composite, 1984 to 2012, accessed July 2017, from https://explorer.earthengine.google.com/#detail/LANDSAT%2FLT5_L1T_ANNUAL_GREENEST_TOA; **4**.Landsat 8 Annual Greenest-Pixel TOA Reflectance Composite, 2013 to 2015, accessed July 2017,from https://explorer.earthengine.google.com/#detail/LANDSAT%2FL C8_L1T_ANNUAL_GREENEST_TOA; **5**.CanMap Postal Code Suite v2015.3. [computer file] Markham: DMTI Spatial Inc., 2015; **6**.Gorelick N, Hancher M, Dixon M, Ilyushchenko S, Thau D, Moore R. Google Earth Engine: Planetary-scale geospatial analysis for everyone. Remote Sensing of Environment. 2017;202:18–27.

### Model results

In the first approach of model fitting, using the General Additive Models for Location Scale and Shape (GAMLSS), 31 distributions were tested for males and females. Based on the Generalized Akaike Information Criteria values (GAIC) the top five distributions were explored further. Different distributions were identified for males (distributions: SHASH, SEP2, SEP1, SHASHo, and SHASHo2) and females (distributions: SEP4, JSU, JSUo, ST1, and ST5). However, differences between GAIC values between distributions was minor (Supplemental Table C1-2). After running different combinations of smoothing techniques (cubic splines, fractional polynomials, and polynomial splines) and degrees of freedom penalties for model complexities (k = 2, 3.84, 5, and 9) the top distributions were identified by GAIC (Supplemental Table C3-4). The Sinh-archsinh – origin link 2 (SHASHo2) for males and the Skew power exponential type 4 (SEP4) for females both smoothed using cubic splines (cs) were moved forwards to compare to quantile regression models from the second modelling approach (linear, polynomials 2-4th order, smoothed quantile regression, and fractional polynomials).

### Cross-validation

The cross-validation showed very minor differences in fit across the seven models (i.e., GAMLSS and 6 quantile regression models). Greater attention was paid to the fit of the 5th percentile representing individuals with the lowest PA levels, as accurately identifying these individuals is of greater importance for PA level promotion and intervention. The models selected were the SHASHo2cs and SEP4cs for males and females (Supplemental Table C5).

### Normative values

**Fig. 2:**
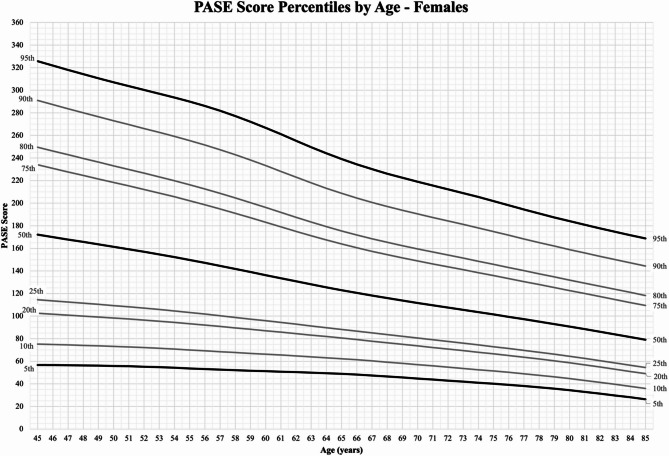
Total PASE score percentiles for females aged 45–85 years old

**Fig. 3:**
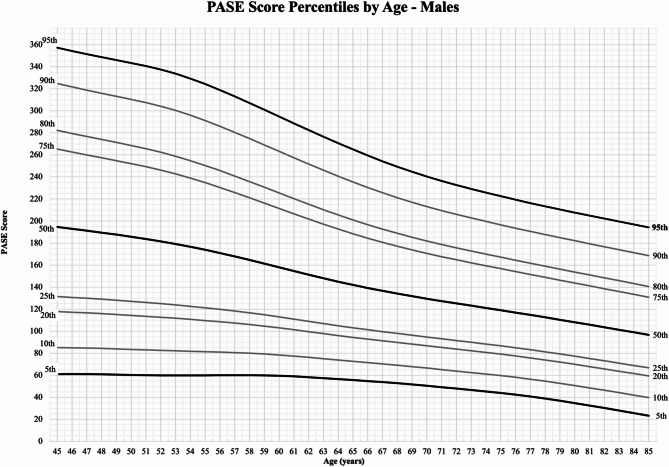
Total PASE score percentiles for males aged 45–85 years old

Normative values for the 5th, 10th, 20th, 25th, 50th, 75th, 80th, 90th, and 95th percentiles for males and females 45 to 85 years old are presented as charts (Figs. 2 and 3) and tables (Supplemental Tables D1-2). Normative values decreased with increasing age; for both males and females the 50th percentiles decreased by approximately 100 points from the age of 45 to 85 years old. Using Figs. 2 and 3, PASE scores for percentile values can be found for any age between 45 and 85. For example, in females (Fig. 2) we can select the age 45 along the x-axis, follow this vertically to the 50th percentile line, then across to the y-axis for a PASE score around 170, whereas the score of an 85-year-old female in the 50th percentile would be around 80. Overall, females had lower normative values across percentiles compared to males of the same age.

### Season stratification

To account for possible seasonal effects on PA behaviour, we performed an analysis stratifying for the season that PA was collected in. For males, almost identical GAMLSS distributions were identified across seasons (Supplemental Table E1). Likewise in females, similar distributions were identified for all but the winter season (Supplemental Table E2). A similar trend was seen when comparing the fit of the seven distributions during cross-validation (Supplemental Table E3). Examining the percentile values across seasons also did not show substantial differences (e.g., for 65-year-old males the 5th percentile scores were 52 for winter, 50 for spring, 50 for summer, and 50 for fall).

## Discussion

Our paper presents the first population-based age- and sex-specific normative values of the PASE, a commonly used and endorsed self-report measure of PA for older adults. The large sample and sampling weights of the CLSA allowed for a robust description of sample demographics to facilitate a broad application and reference for research use with the PASE beyond Canada. This analysis, which demonstrates a consistent age-related decline in PA among healthy adults from age 45 to 85, highlights the importance of considering age-appropriate reference values when assessing PA levels.

Normative values play an important role in monitoring the progress of health characteristics and identifying individuals needing investigation or intervention (e.g., WHO’s child and adolescent growth curves) [7]. By accounting for age and sex in older populations, the normative values presented in this analysis show a downward trend of PASE scores (i.e., PA levels) with increasing age in a sample of ‘healthy’ males and females. Across percentiles, we found that the PASE score for an 85-year-old was close to half of a 45-year-old male or female (e.g., 50th percentile male aged 45 years PASE score of 195 versus PASE score of 97 for 85-year-olds). The preliminary normative values (mean PASE score for males and females across three age groups) provided with the PASE instructional manual (*n* = 396, 1993) also showed declining PA levels with increasing age [30]. Recognizing that age-related decreases occur in healthy older adults (free of mobility limitation and disability) is essential for interpreting changes due to decreased mobility or increased limitation on a person’s PA. The presented normative values also demonstrated a consistent difference between males and females, in line with previous literature showing that women have lower PA levels than men [31]. The demonstrated trends in PA levels highlight the importance of considering healthy variation in PA outcomes across ages.

The use of national and international recommended levels of physical activities (150 min of moderate to vigorous PA) as a cut-off value has allowed for a standardization of monitoring PA levels across countries and populations. However, this one-size-fits-all approach of a single criterion may unfairly penalize certain people. This scenario can be seen with normative values; consider two people with the same PASE score of 172; a 45-year-old female would be in the 50th percentile, whereas an 84-year-old female would be in the 95th percentile. The dichotomous nature of the current standard may not provide enough detail to assess change in PA levels. Normative values allow for monitoring placement across multiple percentiles rather than above or below a singular placement. In addition, they allow for sex specific PA monitoring. Research has demonstrated not only a difference in PA level between males and females but also a difference in response to the same PA level/dose (e.g., females experience greater reductions in cardiovascular and all-cause mortality risk than males) [32]. These differences may suggest that sex-specific activity cut-offs are warranted (e.g., females may require a lower cut-off to experience health benefits than males) and should be considered for future work. Current PA guidelines still provide an excellent goal associated with numerous positive health and wellbeing outcomes; [2] however, we may wish to consider additional metrics when monitoring and promoting PA behaviour as part of healthy aging initiatives.

The creation of normative values for the PASE allows for greater interpretation and applicability of the total score. Scores can now be compared to an age-appropriate reference value and can inform management decisions (e.g., when intervention is needed) based on an average for a ‘healthy’ individual with no PA restrictions [7]. Additionally, normative values offer new opportunities to explore the relationships between PA and health outcomes. For example, they could be used to compare dose-response relationships across age and sex stratifications, trajectories and their relationships to long-term health, or be used as a reference point for healthy aging to flag digressions from expected activity patterns as early indicators of decline. Importantly, our use of the CLSA for creating normative values allowed for the inclusion of detailed descriptive data of the sample (e.g., environmental, health, behaviour, and socioeconomic variables). In addition to generalizing to similar populations (i.e., predominately European descent and higher-income populations), these demographic details provide an opportunity to be used as a comparison based on included features. By providing detailed descriptive data of the sample, we hope to facilitate greater application of these values world-wide.

Notably, despite the often cited need to account for seasonal differences when considering PA levels, normative values across seasons did not vary greatly and did not appear to provide additional information compared to the sex-stratified only values. The lack of variation did align with the majority (70%) of the sample that reported the activity recorded from their previous 7-days was reflective of the past 12 months. Evidence for the seasonal effect on PA has suggested that season may not be as salient across large countries like Canada where there is greater heterogeneity in seasonal weather and temperatures [20]. Therefore, the provided normative values can be compared to PASE scores completed during any season.

### Limitations

There are several limitations to take into consideration for these results. Firstly, the PASE is a self-report measure of PA. Validity studies have shown lower correlations between the PASE and direct measures of PA like accelerometers and pedometers compared to correlations between the PASE and other self-report tools [9, 14, 16]. While this suggests self-report and direct tools may be capturing slightly different constructs, self-reported PA has shown strong associations with important health outcomes and such measures are also widely accessible [33]. Second, the PASE was explicitly developed and validated in populations 65 years and older and not for middle-aged adults [25]. However, as the PASE was designed to be inclusive of a broad range of activities (i.e., including lighter intensities and activities related to employment), it has high face validity for adults in general, and has been validated against other PA questionnaires designed for younger samples (International Physical Activity Questionnaire) [8]. Third, the nature of the Canadian sample must also be acknowledged as a limitation. In particular, Canada is a high-income westernized country; the primarily European descent of participants and the high socioeconomic status (household income and education level) should be considered before generalizing these values to other populations. When comparing the mean PASE score of our analytic sample to values from similarly aged samples from other countries we see some variation but overall similar values (e.g., USA 50–90 years, 155 (SD 81); Italy 55–75 159 (SD 78); and Australia 55–85 years, 168 (SD 81)) [19]. Physical activity data was collected in the CLSA between 2013 and 2015 and may not account for any lasting changes in PA as a result of the pandemic or changes in climate [34]. Finally, there is no one definition of a ‘healthy’ older population. For the presented normative values we defined ‘healthy’, in the context of PA, to be people without restrictions in PA. The WHO defines ‘healthy aging’ as the process of maintaining and improving functional ability with age. By restricting our sample based on mobility and disability criteria (excluding those needing assistance with activities of daily living), we covered the majority of the five components of the WHO’s functional ability domain (ability to meet basic needs, ability to be mobile, and ability to contribute) [1]. For individuals excluded, the percentile values (e.g., 5th, 50th, 95th) may not be appropriate, and creation of normative values for specific population may be warranted (e.g., people who use gait aids). However, the current curves and values could still be a tool for monitoring progress (i.e., increasing PA levels).

## Conclusion

These are the first age and sex specific normative values of the PASE developed for middle-aged and older adults using a large population-based study. The detailed demographic characteristics of the sample provided by the CLSA will allow for broader application of these normative values to other populations with similar characteristics. In addition to PA guideline cut-offs, normative values provide further information for monitoring PA by allowing for more personalized observations that account for variation across ages and sex. The provided values can be used to improve the interpretability of PASE scores and monitoring of PA behaviour with aging.

## Supplementary Information

Below is the link to the electronic supplementary material.

Supplementary Material 1

## Data Availability

Data are available from the Canadian Longitudinal Study on Aging (www.clsa-elcv.ca) for researchers who meet the criteria for access to de-identified CLSA data.
