# Supplementary material for: Normative values for the physical activity scale for the elderly in community-dwelling men and women 45 to 85 years old: an analysis from the CLSA
Source: Int J Behav Nutr Phys Act. 2025 Oct 14;22:127. doi: 10.1186/s12966-025-01820-w (PMC12522245; doi:10.1186/s12966-025-01820-w)
Supplement: Supplementary file 1 — Supplementary Material 1 [file 12966_2025_1820_MOESM1_ESM.pdf]

Table of Contents

APPENDIX A. SAMPLE DEMOGRAPHICS 2

TABLE A1. DEMOGRAPHIC CHARACTERISTICS OF THE UNWEIGHTED SAMPLE 2
TABLE A2: SAMPLE SIZES PER AGE AND SEX GROUP 4

APPENDIX B: INCLUSION/EXCLUSION CRITERIA VALIDATION 5

TABLE B1: INDEPENDENT T-TEST RESULTS PASE SCORES BASED ON SAMPLE CRITERIA FOR FEMALES AND MALES 5

APPENDIX C: RESULTS FROM SEX STRATIFICATION MODEL BUILDING 5

TABLE C1: GENERALIZED AKAIKE INFORMATION CRITERIA (GAIC) VALUES FOR *REALLINE* AND *REALPLUS* OF DISTRIBUTIONS TESTED FOR MALES 5
TABLE C2: GENERALIZED AKAIKE INFORMATION CRITERIA (GAIC) VALUES FOR *REALLINE* AND *REALPLUS* OF DISTRIBUTIONS TESTED FOR FEMALES 6
TABLE C3: RANKING OF TOP COMBINATIONS BY THE GENERALIZED AKAIKE INFORMATION CRITERIA OF DISTRIBUTIONS AND SMOOTHING TECHNIQUES IN **MALES** USING DEGREES OF PENALIZATION FOR MODEL COMPLEXITY 7
TABLE C4: RANKING OF TOP COMBINATIONS BY THE GENERALIZED AKAIKE INFORMATION CRITERIA OF DISTRIBUTIONS AND SMOOTHING TECHNIQUES IN **FEMALES** USING DEGREES OF PENALIZATION FOR MODEL COMPLEXITY 8
TABLE C5: CROSS-VALIDATION RESULTS 8

APPENDIX D: FINAL PERCENTILES TABLES FOR FEMALES AND MALES 9

TABLE D1: PERCENTILES BY AGE – FEMALES 9
TABLE D2: PERCENTILES BY AGE – MALES 10

APPENDIX E: RESULTS FROM SEX AND SEASON STRATIFICATION MODEL BUILDING 11

TABLE E1: GENERALIZED AKAIKE INFORMATION CRITERIA (GAIC) VALUES FOR *REALLINE* AND *REALPLUS* OF DISTRIBUTIONS TESTED 11
TABLE E2: GENERALIZED AKAIKE INFORMATION CRITERIA (GAIC) VALUES FOR *REALLINE* AND *REALPLUS* OF DISTRIBUTIONS TESTED 12
TABLE E3: CROSS-VALIDATION RESULTS 13

Appendix A. Sample demographics

Table A1. Demographic characteristics of the unweighted sample

	Sample		
	Total n=36,701	Male n=19,379 (53%)	Female n=17,322 (47%)
**Age**	61.63 (9.94)	62.14 (10.10)	61.07 (9.76)
**Cultural/racial background**			
European	34,837 (95%)	18,316 (95%)	16,521 (95%)
Non-European	1,397 (4%)	803 (4%)	594 (3%)
Multiple origins	429 (1%)	232(1%)	197 (1%)
**Marital status**			
In a relationship	26,162 (71%)	15,271 (79%)	10,891 (63%)
Widowed	2,987 (8%)	859 (4%)	2,201 (13%)
Divorced/separated	4,361 (12%)	1,687 (9%)	2,674 (15%)
Single	2,702 (7%)	1,297 (7%)	1,405 (8%)
**Had a fall in the previous 12 months** (Yes)	3,550 (10%)	1,665 (9%)	1,885 (5%)
**Diagnosed with a chronic medical condition**			
Cancer	5,006 (14%)	2,601 (13%)	2,405 (14%)
Cardiovascular	15,116 (41%)	8,787 (24%)	6,329 (37%)
Diabetes	5,392 (15%)	3,325 (17%)	2,067 (12%)
Mental health	6,116 (17%)	2,554 (13%)	3,562 (21%)
Musculoskeletal	17,580 (48%)	8,432 (44%)	9,148 (53%)
Neurological	1,923 (5%)	1,112 (6%)	811 (5%)
Respiratory	5,237 (14%)	2,490 (13%)	2,747 (16%)
Vision	9,375 (26%)	4,715 (24%)	4,660 (27%)
**Number of medications**			
None	7,493 (20%)	4,302 (22%)	3,191 (18%)
One	5,881 (16%)	3,083 (16%)	2,798 (16%)
Two	4,974 (14%)	2,609 (13%)	2,365 (14%)
3+	18,263 (50%)	9,334 (48%)	8,929 (52%)
**Smoking behaviour**			
Current smoker	3,045 (8%)	1,583 (8%)	1,462 (9%)
Former smoker	21,918 (60%)	12,365 (64%)	9,553 (55%)
Never smoked	11,549 (32%)	5,326 (28%)	6,223 (36%)
**Self-reported general health**			
Excellent	8,225 (22%)	4,084 (21%)	4,141 (24%)
Very good	15,914 (43%)	8,113 (42%)	7,801 (45%)
Good	10,113 (28%)	5,719 (30%)	4,394 (25%)
Fair	2,129 (6%)	1,274 (7%)	855 (5%)
Poor	297 (<1%)	176 (<1%)	121 (<1%)

	Sample		
	Total n=36,701	Male n=19,379 (53%)	Female n=17,322 (47%)
**Education**			
Less than secondary school graduation	2,013 (5%)	1,058 (5%)	955 (6%)
Secondary school graduation	3,875 (11%)	1,879 (10%)	1,966 (10%)
Some post-secondary education	2,577 (7%)	1,339 (7%)	1,238 (6%)
Post-secondary degree/diploma	28,165 (77%)	15,057 (78%)	13,208 (68%)
**Household income**			
less than $20,000	1,387 (4%)	538 (3%)	849 (5%)
$20,000 or more, but less than $50,000	7,551 (21%)	3,443 (18%)	4,108 (24%)
50,000 or more, but less than $100,000	12,621 (34%)	6,893 (36%)	5,728 (33%)
$100,000 or more, but less than $150,000	7,009 (19%)	4,049 (21%)	2,960 (17%)
$150,000 or more	6,026 (16%)	3,623 (19%)	2,403 (14%)
Missing	2,107 (6%)	833 (4%)	1,274 (7%)
**Type of dwelling**			
House (detached, semi-detached, duplex or townhouse)	31,008 (85%)	16,662 (86%)	14,343 (83%)
Apartment or condominium	5,378 (15%)	2,569 (13%)	2,812 (16%)
Other	307 (<1%)	143 (1%)	164 (1%)
**Classified as living in rural area**	5,067 (14%)	2,659 (14%)	2,408 (14%)
**Greenspace** (Normalized difference vegetation index)			
Mean of NDVI within 500 m	0.42 (0.12)	0.42 (0.12)	0.41 (0.12)
**Neighborhood safety**			
Local area is kept very clean (agreed)	35,195 (96%)	18,564 (96%)	16,631 (96%)
People would be **afraid** to walk alone after dark in local area (agreed)	3,396 (9%)	1,302 (7%)	2,094 (12%)
Vandalism or graffiti are a big problem in local area (agreed)	2,068 (6%)	1,077 (6%)	991 (6%)
**Physical activity (Total PASE score)**	150.81 (74.88)	158.95 (77.69)	141.70 (70.51)
**Physical represent last 12 months**			
Agreed	24,996 (68%)	13,681 (71%)	11,315 (65%)
Neither	779 (2%)	405 (2%)	374 (2%)
Disagreed	10,805 (29%)	5,227 (27%)	5,577 (32%)
**Season of physical activity measurement**			
Winter (Jan-Mar)	6,659 (18%)	3,514 (18%)	3,145 (18%)
Spring (Apr-Jun)	10,262 (28%)	5,445 (28%)	4,817 (28%)
Summer (Jul-Sept)	10,688 (29%)	5,672 (29%)	5,016 (29%)
Autumn (Oct-Dec)	9,092 (25%)	4,748 (25%)	4,344 (25%)

Missing values were only recorded when greater than or equal to 5% of data were missing.
Footnote: NDVI metrics, indexed to DMTI Spatial Inc. postal codes, were provided by CANUE (Canadian Urban Environmental Health Research Consortium).

	Sample		
	Total n=36,701	Male n=19,379 (53%)	Female n=17,322 (47%)
1.USGS Landsat 5 TM TOA Reflectance (Orthorectified), 1984 to 2011, accessed July 2017, from https://explorer.earthengine.google.com/#detail/LANDSAT%2FL T5_L1T_TOA.<br>2.USGS Landsat 8 TOA Reflectance (Orthorectified), 2013 to 2017, accessed July 2017, from https://explorer.earthengine.google.com/#detail/LANDSAT%2FL C8_L1T_TOA.<br>3.Landsat 5 TM Annual Greenest-Pixel TOA Reflectance Composite, 1984 to 2012, accessed July 2017, from https://explorer.earthengine.google.com/#detail/LANDSAT%2FL T5_L1T_ANNUAL_GREENEST_TOA.<br>4.Landsat 8 Annual Greenest-Pixel TOA Reflectance Composite, 2013 to 2015, accessed July 2017,from https://explorer.earthengine.google.com/#detail/LANDSAT%2FL C8_L1T_ANNUAL_GREENEST_TOA.<br>5.CanMap Postal Code Suite v2015.3. [computer file] Markham: DMTI Spatial Inc., 2015.<br>6.Gorelick N, Hancher M, Dixon M, Ilyushchenko S, Thau D, Moore R. Google Earth Engine: Planetary-scale geospatial analysis for everyone. Remote Sensing of Environment. 2017;202:18-27.			

Table A2: Sample size per age and sex group

**Age**	Total	Male	Female	**Age**	Total	Male	Female	**Age**	Total	Male	Female
**44**	5	1	4	**59**	1285	665	620	**74**	528	251	277
**45**	449	224	225	**60**	1331	663	668	**75**	744	315	429
**46**	723	366	357	**61**	1261	559	702	**76**	761	326	435
**47**	842	424	418	**62**	1317	583	734	**77**	712	305	407
**48**	919	469	450	**63**	1349	651	698	**78**	547	238	309
**49**	1172	560	612	**64**	1260	555	705	**79**	581	244	337
**50**	1252	597	655	**65**	1199	593	606	**80**	461	199	262
**51**	1296	642	654	**66**	1036	493	543	**81**	383	147	236
**52**	1299	654	645	**67**	951	447	504	**82**	331	128	203
**53**	1380	687	693	**68**	994	445	549	**83**	255	101	154
**54**	1478	724	754	**69**	891	381	510	**84**	196	84	112
**55**	1083	558	525	**70**	793	359	434	**85**	130	57	73
**56**	1094	579	515	**71**	737	318	419	**86**	20	7	13
**57**	1141	573	568	**72**	652	281	371	**87**	1	0	1
**58**	1277	610	667	**73**	583	257	326	**88**	2	2	0

Appendix B: Inclusion/exclusion criteria validation

Table B1: Independent t-test results PASE scores based on sample criteria for females and males.

	**Met inclusion criteria** Mean (SD)	**Did not meet inclusion criteria** Mean (SD)	**Significantly different** t-value (DF) p-value
**Total**	150.81 (74.88)	110.12 (67.73)	t=52.861 (18,320), p<0.001
**Females**	141.70 (70.51)	105.40 (64.11)	t=38.279 (13,346), p<0.001
**Males**	158.95 (77.69)	118.64 (73.06)	t=30.511 (5,463.4), p<0.001

Appendix C: Results from sex stratification model building

Table C1: Generalized Akaike Information Criteria (GAIC) values for *realline* and *realplus* of distributions tested for males

**Distribution name (acronym)**	**Male GAIC values**
Sinh-archsinh – identity link (**SHASH**)	**221923.33**
Skew power exponential type 2 (**SEP2**)	**221926.17**
Skew power exponential type 1 **(SEP1**)	**221937.9**
Sinh-archsinh – origin link (**SHASHo**)	**221947.04**
Sinh-archsinh – origin link 2 (**SHASHo2**)	**221947.04**
Skew t distribution type 1 (**ST1**)	221951.17
Skew t distribution type 2 (**ST2**)	221951.17
Skew power exponential type 3 (**SEP3**)	222000.44
Skew normal type 2 (**SN2**)	222005.02
Skew t distribution type 3 (**ST3**)	222006.96
Skew t distribution – reparametrized (**SST**)	222006.96
Skew power exponential type 4 (**SEP4**)	222020.93
Johnson's Su – identify link **(JSU**)	222025.9
Johnson's Su – origin link (**JSUo**)	222025.93
Skew t distribution type 5 (**ST5**)	222062.45
Reverse Gumbel (**RG**)	222203.24
Ex-Gaussian (**exGAUS**)	222339.62
Skew t distribution type 4 (**ST4**)	222966.67
Generalized t (**GT**)	223586.41
T-family (**TF**)	223633.85
T-family 2 (**TF2**)	223633.85
Power exponential (**PE**)	223684.77
Power exponential 2 (**PE2**)	223684.77
Normal (**NO**)	223701.52
Skew normal type 1 (**SN1**)	223703.52
Logistic (**LO**)	223825.62
Normal exponential t (**NET**)	224597.09
Gumbel (**GU**)	231015.55
Exponential (**EXP**)	235207.66
Pareto – identify link (**PARETO2**)	235209.67
Pareto – origin link (**PARETO2o**)	235209.74

Green = top 5

Table C2: Generalized Akaike Information Criteria (GAIC) values for *realline* and *realplus* of distributions tested for females

Distribution name (acronym)	Female GAIC values
Skew power exponential type 4 (**SEP4**)	**194251.45**
Johnson's Su – identify link **(JSU**)	**194267.14**
Johnson's Su – origin link (**JSUo**)	**194267.15**
Skew t distribution type 1 (**ST1**)	**194305.75**
Skew t distribution type 5 (**ST5**)	**194306.28**
Skew t distribution type 2 (**ST2**)	194307.16
Reverse Gumbel (**RG**)	194323.8
Skew power exponential type 1 **(SEP1**)	194331.85
Sinh-archsinh – identity link (**SHASH**)	194332.21
Skew power exponential type 2 (**SEP2**)	194334.78
Skew t distribution type 3 (**ST3**)	194366.01
Skew t distribution – reparametrized (**SST**)	194366.01
Sinh-archsinh – origin link (**SHASHo**)	194399.65
Sinh-archsinh – origin link 2 (**SHASHo2**)	194399.65
Skew power exponential type 3 (**SEP3**)	194404.61
Skew normal type 2 (**SN2**)	194420.56
Ex-Gaussian (**exGAUS**)	194560.91
Skew t distribution type 4 (**ST4**)	195215.73
Generalized t (**GT**)	196061.99
T-family (**TF**)	196155.51
T-family 2 (**TF2**)	196155.51
Logistic (**LO**)	196236.05
Power exponential (**PE**)	196359.57
Power exponential 2 (**PE2**)	196359.57
Normal (**NO**)	196595.63
Skew normal type 1 (**SN1**)	196597.63
Normal exponential t (**NET**)	196780.79
Exponential (**EXP**)	206261.77
Pareto – identify link (**PARETO2**)	206263.8
Pareto – origin link (**PARETO2o**)	206263.88
Gumbel (**GU**)	206433.16

Green = top 5

Table C3: Ranking of top combinations by the Generalized Akaike Information Criteria of distributions and smoothing techniques in **males** using degrees of penalization for model complexity

Distribution	Smoothing technique	Degrees of freedom	Degree of penalty for model complexity							
			k=2		k=3.84		k=5		k=9	
			Ranking	GAIC	Ranking	GAIC	Ranking	GAIC	Ranking	GAIC
**SHASHo2**	**cs**	24.0	1	217934.95	1	217979.11	1	218006.95	1	218102.96
**SHASH**	**cs**	24.0	2	217936.03	2	217980.20	2	218008.04	2	218104.05
**SHASHo**	**fp**	24.0	3	217963.19	3	218007.35	3	218035.19	3	218131.19
**SEP2**	**cs**	24.0	4	217965.11	4	218009.27	4	218037.11	4	218133.11
**SHASH**	**fp**	24.0	5	217981.07	5	218025.23	5	218053.07	5	218149.07
**SEP2**	**fp**	24.0	6	217995.72	6	218039.88	6	218067.72	6	218163.71
**SEP1**	**fp**	24.0	7	217998.44	7	218042.60	7	218070.44	7	218166.44
**SHASHo2**	**fp**	24.0	8	218003.18	8	218047.34	8	218075.18	8	218171.18
**SHASH**	**pb**	17.6	9	218058.25	9	218090.65	9	218111.08	9	218181.51
**SHASHo2**	**pb**	17.1	10	218073.36	11	218104.83	11	218124.66	11	218193.06
**SHASHo**	**pb**	17.1	11	218073.78	12	218105.22	12	218125.04	12	218193.37
**SEP1**	**pb**	15.2	12	218075.75	10	218103.70	10	218121.32	10	218182.08
**SEP1**	**cs**	24.0	13	218249.73	13	218293.89	13	218321.73	13	218417.74
**SEP2**	**pb**	18.4	14	218730.57	14	218764.49	14	218785.88	15	218859.63
**NO**	**pb**	15.6	15	218768.37	15	218797.08	15	218815.19	16	218877.62
**NO**	**cs**	12.0	16	218774.00	16	218796.08	16	218810.00	14	218858.00
**NO**	**fp**	12.0	17	218851.16	17	218873.24	17	218887.16	17	218935.16

Table C4: Ranking of top combinations by the Generalized Akaike Information Criteria of distributions and smoothing techniques in **females** using degrees of penalization for model complexity

Distribution	Smoothing technique	Degrees of freedom	Degree of penalty for model complexity							
			k=2		k=3.84		k=5		k=9	
			Ranking	GAIC	Ranking	GAIC	Ranking	GAIC	Ranking	GAIC
**SEP4**	**cs**	20.0	1	200685.60	1	200722.41	1	200745.61	1	200825.61
**SEP4**	**fp**	20.0	2	200723.51	2	200760.31	2	200783.51	3	200863.51
**JSU**	**pb**	13.9	3	200744.36	3	200769.89	3	200785.99	2	200841.50
**SEP4**	**pb**	16.1	4	200766.75	4	200797.23	4	200816.44	4	200882.71
**JSUo**	**cs**	20.0	5	200779.68	5	200816.49	5	200839.69	5	200919.69
**JSUo**	**fp**	20.0	6	200788.30	6	200825.10	6	200848.30	6	200928.30
**ST5**	**fp**	20.0	7	200820.59	7	200857.39	7	200880.59	8	200960.59
**ST1**	**fp**	20.0	8	200829.59	8	200866.39	8	200889.59	10	200969.59
**ST1**	**pb**	11.4	9	200875.78	9	200896.79	9	200910.03	7	200955.68
**JSUo**	**pb**	10.2	10	200894.44	10	200913.24	10	200925.09	9	200965.95
**ST5**	**pb**	10.0	11	202508.46	11	202526.86	11	202538.46	11	202578.46
**ST5**	**cs**	20.0	12	200921.75	12	200958.56	12	200981.76	12	201061.77
**NO**	**pb**	16.0	13	202434.58	13	202464.06	13	202482.64	13	202546.72
**NO**	**cs**	10.0	14	202508.46	14	202526.86	14	202538.46	14	202578.46
**NO**	**fp**	10.0	15	202554.89	15	202573.29	15	202584.89	15	202624.89
**JSU**	**fp**	20.0	16	2962644.22	16	2962681.02	16	2962704.22	16	2962784.22
**JSU**	**cs**	20.0	17	3090191.08	17	3090227.88	17	3090251.08	17	3090331.07

Table C5: Cross-validation results

	Average percent of observed participants above and below thresholds from cross-validation									
	Sex	Threshold for comparison of observed vs. expected	GAMLSS		Quantile regression					
					Age only	Polynomial models			Smoothed using "*arqss*"	Fractional polynomials
			SHASHo2 cs	SEP4cs		Second order	Third order	Fourth order		
**All Year**	**Males**	<5%	**5.01**		4.87	4.87	4.80	4.80	4.09	4.90
		<50%	**50.36**		51.66	51.66	51.76	51.74	51.48	51.73
		>95%	**4.59**		4.19	4.19	4.17	4.18	4.24	4.19
	**Females**	<5%		**5.10**	4.84	4.84	4.87	4.86	4.81	4.88
		<50%		**49.31**	49.18	49.18	49.20	49.16	49.21	49.18
		>95%		**4.50**	5.18	5.18	5.20	5.20	5.18	5.18

Appendix D: Final percentiles tables for females and males

Table D1: Percentiles by age – Females

Age (years)	Female Total PASE Score Percentiles								
	Percentiles								
	5th	10th	20th	25th	50th	75th	80th	90th	95th
**45**	57	75	102	115	**172**	234	250	291	326
**46**	57	75	102	114	**170**	231	246	287	322
**47**	56	74	101	112	**168**	228	243	284	318
**48**	56	74	100	111	**166**	225	240	280	314
**49**	56	74	99	110	**164**	221	236	276	311
**50**	56	73	98	109	**161**	218	233	273	307
**51**	56	73	97	108	**159**	215	230	270	304
**52**	55	72	96	107	**157**	212	227	266	300
**53**	55	72	95	106	**155**	209	223	263	297
**54**	54	71	94	105	**152**	206	220	259	294
**55**	54	70	93	103	**150**	202	216	255	290
**56**	53	69	92	102	**147**	199	213	252	286
**57**	53	68	91	100	**145**	195	209	247	282
**58**	52	68	90	99	**142**	191	205	243	277
**59**	52	67	88	97	**139**	187	200	238	272
**60**	51	66	87	96	**136**	183	196	233	267
**61**	51	66	86	94	**134**	179	192	228	261
**62**	50	65	85	93	**131**	175	188	223	255
**63**	50	64	83	91	**128**	171	183	218	250
**64**	49	63	82	90	**125**	167	179	213	244
**65**	49	62	81	88	**123**	164	175	209	239
**66**	48	61	79	87	**121**	161	172	204	234
**67**	47	60	78	85	**118**	157	169	201	230
**68**	47	59	76	84	**116**	154	165	197	226
**69**	46	58	75	82	**114**	152	162	194	222
**70**	45	57	74	81	**112**	149	160	190	219
**71**	44	56	72	79	**110**	146	157	187	216
**72**	43	55	71	77	**108**	144	154	184	212
**73**	42	54	69	76	**105**	141	151	181	209
**74**	41	52	68	74	**103**	138	149	178	205
**75**	40	51	67	73	**101**	136	146	175	202
**76**	39	50	65	71	**99**	133	143	172	198
**77**	38	49	64	70	**97**	131	140	168	194
**78**	37	48	62	68	**95**	128	137	165	191
**79**	36	46	60	66	**93**	125	135	162	187
**80**	34	45	59	64	**91**	123	132	159	184
**81**	33	43	57	63	**88**	120	129	156	181
**82**	31	41	55	61	**86**	117	126	153	178
**83**	30	40	53	59	**84**	115	124	150	175
**84**	28	38	51	56	**81**	112	121	147	172
**85**	26	36	49	54	**79**	109	118	144	169

Table D2: Percentiles by age – Males

Age (years)	Male Total PASE Score Percentiles								
	Percentiles								
	5th	10th	20th	25th	50th	75th	80th	90th	95th
**45**	61	85	118	131	**195**	265	282	325	357
**46**	61	85	117	131	**193**	263	279	322	354
**47**	61	85	117	130	**191**	260	277	319	351
**48**	61	84	116	129	**189**	257	274	316	349
**49**	61	84	115	128	**188**	255	271	313	346
**50**	60	83	114	127	**186**	252	269	310	343
**51**	60	83	114	126	**184**	249	266	307	341
**52**	60	83	113	125	**182**	246	262	304	337
**53**	60	82	112	124	**179**	243	259	300	334
**54**	60	82	111	123	**177**	239	255	296	329
**55**	60	81	110	121	**174**	235	250	291	324
**56**	60	81	109	120	**171**	230	246	286	319
**57**	60	81	107	118	**168**	226	241	281	313
**58**	60	80	106	117	**165**	221	236	275	307
**59**	60	79	105	115	**161**	216	231	269	301
**60**	60	78	103	113	**158**	211	225	263	295
**61**	59	77	101	111	**155**	207	220	257	289
**62**	58	76	100	109	**151**	202	215	252	283
**63**	57	75	98	107	**148**	197	210	246	277
**64**	57	74	96	105	**145**	193	206	241	271
**65**	56	73	94	103	**142**	188	201	235	265
**66**	55	71	93	101	**139**	184	197	230	259
**67**	54	70	91	100	**137**	181	193	226	254
**68**	53	69	90	98	**134**	177	189	221	249
**69**	52	68	88	96	**132**	174	185	217	245
**70**	51	67	87	95	**130**	171	182	213	240
**71**	49	65	85	93	**127**	168	179	209	236
**72**	48	64	84	92	**125**	165	176	206	233
**73**	47	62	82	90	**123**	162	173	203	229
**74**	45	61	81	88	**121**	159	170	200	226
**75**	44	60	79	87	**119**	157	167	197	223
**76**	42	58	78	85	**117**	154	164	194	219
**77**	41	56	76	83	**115**	151	162	191	216
**78**	39	55	74	81	**113**	149	159	188	213
**79**	37	53	72	79	**110**	146	156	185	210
**80**	35	51	70	77	**108**	144	154	182	208
**81**	33	49	68	75	**106**	141	151	179	205
**82**	30	46	66	73	**104**	139	148	177	202
**83**	28	44	64	71	**101**	136	146	174	200
**84**	26	42	62	69	**99**	133	143	171	197
**85**	23	40	59	67	**97**	131	141	169	194

Appendix E: Results from sex and season stratification model building

Table E1: Generalized Akaike Information Criteria (GAIC) values for *realline* and *realplus* of distributions tested

Distribution name (acronym)	GAIC values for Males			
	Winter	Spring	Summer	Fall
Normal (**NO**)	40401.00	62999.51	65363.10	54889.66
Gumbel (**GU**)	41686.41	64808.65	67687.93	56741.52
Reverse Gumbel (**RG**)	40059.52	62589.10	64980.40	54518.22
Logistic (**LO**)	40423.31	196236.05	65362.15	54922.69
Normal exponential t (**NET**)	40553.57	63061.73	65598.01	55098.63
T-family (**TF**)	40391.76	62997.39	65325.80	54869.70
T-family 2 (**TF2**)	40391.76	62997.39	65325.80	54869.70
Power exponential (**PE**)	40401.29	63000.73	65340.95	54889.02
Power exponential 2 (**PE2**)	40401.29	63000.73	65340.95	54889.02
Skew normal type 1 (**SN1**)	40403.00	63001.51	65365.10	54891.66
Skew normal type 2 (**SN2**)	40032.39	62525.32	64926.81	54469.37
Ex-Gaussian (**exGAUS**)	40100.30	62636.19	64976.73	54562.11
Sinh-archsinh – identity link (**SHASH**)	**40012.82**	**62500.53**	**64899.60**	**54452.51**
Sinh-archsinh – origin link (**SHASHo**)	**40016.48**	**62508.10**	64907.86	**54458.05**
Sinh-archsinh – origin link 2 (**SHASHo2**)	**40016.49**	**62508.10**	64907.86	**54458.05**
Johnson's Su – identify link **(JSU**)	40045.42	62544.02	64910.44	54478.05
Johnson's Su – origin link (**JSUo**)	40045.47	62544.02	64910.44	54478.05
Skew power exponential type 1 **(SEP1**)	**40020.62**	**62506.44**	**64903.36**	**54455.73**
Skew power exponential type 2 (**SEP2**)	**40018.28**	**62503.39**	**64901.38**	**54452.66**
Skew power exponential type 3 (**SEP3**)	40029.52	62527.28	64920.53	54470.49
Skew power exponential type 4 (**SEP4**)	40047.96	62535.87	64910.64	54478.88
Skew t distribution type 1 (**ST1**)	40022.22	62513.86	**64903.89**	54459.67
Skew t distribution type 2 (**ST2**)	40022.22	62513.86	**64903.90**	54459.67
Skew t distribution type 3 (**ST3**)	40034.39	62527.32	64925.63	54471.17
Skew t distribution type 4 (**ST4**)	40256.23	62826.16	65125.82	54713.75
Skew t distribution type 5 (**ST5**)	40054.18	62556.28	64916.34	54487.54
Skew t distribution – reparametrized (**SST**)	40034.39	62527.32	64925.63	54471.17
Generalized t (**GT**)	40375.89	62995.12	65325.67	54837.07
Pareto – identify link (**PARETO2**)	42301.44	66079.31	69144.43	57683.73
Pareto – origin link (**PARETO2o**)	42301.44	66079.28	69144.40	57683.73
Exponential (**EXP**)	42299.44	66077.28	69142.40	57681.73

Green = top 5

Table E2: Generalized Akaike Information Criteria (GAIC) values for *realline* and *realplus* of distributions tested

Distribution name (acronym)	GAIC values for Females			
	Winter	Spring	Summer	Fall
Normal (**NO**)	35501.81	54519.16	56780.97	49715.34
Gumbel (**GU**)	36695.82	57465.28	59906.61	51995.31
Reverse Gumbel (**RG**)	35100.30	**53886.65**	56160.64	**49089.54**
Logistic (**LO**)	35521.25	54394.31	56630.51	49615.23
Normal exponential t (**NET**)	35644.33	54550.62	56780.75	49734.97
T-family (**TF**)	35491.65	54373.72	56613.83	49598.59
T-family 2 (**TF2**)	35491.65	54373.72	56613.83	49598.59
Power exponential (**PE**)	35502.79	54434.17	56670.36	49655.10
Power exponential 2 (**PE2**)	35502.79	54434.17	56670.36	49655.10
Skew normal type 1 (**SN1**)	35503.81	54521.16	56782.97	49717.34
Skew normal type 2 (**SN2**)	35060.01	53932.57	56216.42	49112.11
Ex-Gaussian (**exGAUS**)	35159.94	53945.41	56192.14	49162.40
Sinh-archsinh – identity link (**SHASH**)	**35049.56**	53899.48	56164.48	49099.39
Sinh-archsinh – origin link (**SHASHo**)	**35054.30**	53923.10	56188.48	49116.27
Sinh-archsinh – origin link 2 (**SHASHo2**)	**35054.30**	53923.10	56188.48	49116.27
Johnson's Su – identify link **(JSU**)	35092.42	**53872.51**	**56142.84**	**49078.70**
Johnson's Su – origin link (**JSUo**)	35092.45	**53872.52**	**56142.84**	**49078.70**
Skew power exponential type 1 **(SEP1**)	**35055.78**	53902.58	56172.15	49094.88
Skew power exponential type 2 (**SEP2**)	**35056.37**	53904.75	56177.56	49094.97
Skew power exponential type 3 (**SEP3**)	35061.94	53926.35	56198.88	49113.14
Skew power exponential type 4 (**SEP4**)	35074.79	**53872.01**	**56145.35**	**49068.19**
Skew t distribution type 1 (**ST1**)	35058.20	53890.62	**56157.32**	**49089.99**
Skew t distribution type 2 (**ST2**)	35058.20	53891.28	56158.40	49090.23
Skew t distribution type 3 (**ST3**)	35062.01	53910.93	56181.79	49104.83
Skew t distribution type 4 (**ST4**)	35347.31	54113.55	56344.29	49344.36
Skew t distribution type 5 (**ST5**)	35108.08	**53881.03**	**56145.40**	49092.63
Skew t distribution – reparametrized (**SST**)	35062.01	53910.93	56181.79	49104.83
Generalized t (**GT**)	35460.58	54356.17	56606.71	49564.00
Pareto – identify link (**PARETO2**)	37060.78	66079.31	59962.43	51804.15
Pareto – origin link (**PARETO2o**)	37060.79	66079.28	59962.40	51804.14
Exponential (**EXP**)	37058.78	66077.28	59960.40	51802.14

Green = top 5

Table E3: Cross-validation results

	Average percent of observed participants above and below thresholds from cross-validation									
	Sex	Threshold for comparison of observed vs. expected	GAMLSS		Quantile regression					
					Age only	Polynomial models			Smoothed using “*arqss*”	Fractional polynomials
			SHASHo2cs	SEP4cs		Second order	Third order	Fourth order		
All Year	Males	<5%	**5.01**		4.87	4.87	4.80	4.80	4.09	***4.90***
		<50%	**50.36**		51.66	51.66	51.76	51.74	51.48	***51.73***
		>95%	**4.59**		4.19	4.19	4.17	4.18	4.24	***4.19***
	Females	<5%		**5.10**	4.84	4.84	4.87	4.86	4.81	***4.88***
		<50%		**49.31**	49.18	49.18	49.20	49.16	49.21	***49.18***
		>95%		**4.50**	5.18	5.18	5.20	5.20	5.18	***5.18***
Jan - Mar	Males	<5%	**5.19**		5.50	5.50	5.51	5.53	5.38	5.44
		<50%	**50.66**		51.01	51.01	50.88	50.95	50.94	50.96
		>95%	**4.75**		4.40	4.40	4.44	4.55	4.44	4.47
	Females	<5%		4.69	4.91	4.91	4.88	4.93	4.66	**4.95**
		<50%		49.92	49.19	49.19	49.20	49.34	49.12	**49.36**
		>95%		4.16	5.18	5.18	5.17	5.16	5.02	**5.06**
Apr - Jun	Males	<5%	**5.19**		5.50	5.50	5.51	5.53	5.38	5.44
		<50%	**50.66**		51.01	51.01	50.88	50.95	50.94	50.96
		>95%	**4.75**		4.40	4.40	4.44	4.55	4.44	4.47
	Females	<5%		**5.02**	4.73	4.73	4.74	4.72	4.61	4.71
		<50%		**47.62**	48.37	48.37	48.41	48.34	48.56	48.50
		>95%		**4.75**	5.43	5.43	5.44	5.44	5.30	5.39
Jul - Sept	Males	<5%	5.54		4.62	4.62	4.78	**4.81**	4.60	4.74
		<50%	50.35		51.88	51.88	51.74	**51.84**	51.94	51.84
		>95%	4.77		4.50	4.50	4.52	**4.54**	4.46	4.51
	Females	<5%		5.26	5.17	5.17	5.17	5.20	**5.04**	5.20
		<50%		49.37	49.77	49.77	49.73	49.76	**49.81**	49.81
		>95%		5.08	5.26	5.26	5.31	5.31	**5.27**	5.27
Oct - Dec	Males	<5%	**5.19**		5.54	5.54	5.54	5.50	5.53	4.63
		<50%	**50.16**		51.54	51.54	51.56	51.59	51.46	51.38
		>95%	**4.48**		4.62	4.62	4.56	4.55	4.57	4.63
	Females	<5%		4.49	4.90	4.90	**4.94**	4.84	4.81	4.91
		<50%		47.97	48.77	48.77	**48.77**	48.77	49.04	48.83
		>95%		5.05	5.25	5.25	**5.29**	5.37	5.15	5.31
